# Fast Constant-Time Modular Inversion over $F_{p}$ Resistant to Simple Power Analysis Attacks for IoT Applications

**DOI:** 10.3390/s22072535

**Published:** 2022-03-25

**Authors:** Anissa Sghaier, Medien Zeghid, Chiraz Massoud, Hassan Yousif Ahmed, Abdellah Chehri, Mohsen Machhout

**Affiliations:** 1 Electronics and Micro-Electronics Laboratory, Faculty of Sciences, University of Monastir, Monastir 5000, Tunisia; sghaier.anissa@gmail.com (A.S.); medien.zeghid@fsm.rnu.tn (M.Z.); massoud.chiraz@hotmail.fr (C.M.); machhout@yahoo.fr (M.M.); 2 Electrical Engineering Department, College of Engineering at Wadi Aldawaser, Prince Sattam Bin Abdulaziz University, Wadi Aldawaser 11991, Saudi Arabia; hassanuofg@gmail.com; 3 Department of Applied Sciences, University of Quebec in Chicoutimi (UQAC), Chicoutimi, QC G7H 2B1, Canada

**Keywords:** IoT, PKCs, prime field, modular inversion, BEEA, modular addition and subtraction, SPA, ADP, FPGA

## Abstract

The advent of the Internet of Things (IoT) has enabled millions of potential new uses for consumers and businesses. However, with these new uses emerge some of the more pronounced risks in the connected object domain. Finite fields play a crucial role in many public-key cryptographic algorithms (PKCs), which are used extensively for the security and privacy of IoT devices, consumer electronic equipment, and software systems. Given that inversion is the most sensitive and costly finite field arithmetic operation in PKCs, this paper proposes a new, fast, constant-time inverter over prime fields $F_{p}$ based on the traditional Binary Extended Euclidean (BEE) algorithm. A modified BEE algorithm (MBEEA) resistant to simple power analysis attacks (SPA) is presented, and the design performance area-delay over $F_{p}$ is explored. Furthermore, the BEE algorithm, modular addition, and subtraction are revisited to optimize and balance the MBEEA signal flow and resource utilization efficiency. The proposed MBEEA architecture was implemented and tested on Xilinx FPGA Virtex #5, #6, and #7 devices. Our implementation over $F_{p}$ (length of *p* = 256 bits) with 2035 slices achieved one modular inversion in only 1.12 μs on Virtex-7. Finally, we conducted a thorough comparison and performance analysis to demonstrate that the proposed design outperforms the competing designs, i.e., has a lower area-delay product (ADP) than the reported inverters.

## 1. Introduction

The IoT encompasses the idea that everyday objects can be connected to the Internet and interfere with each other. Thus, these objects are capable of exchanging and storing data. The concept of IoT is to create a link between the real world and the digital world. However, several high-profile incidents have highlighted the vulnerability of IoT security because a common device was used to penetrate a larger network.

Connected objects are being used more and more, especially with the new connected cities projects. This increases the security risks of the IoT, especially when there is no monitoring or management of the devices. For example, any security breach in medical devices in healthcare applications (wearable or implantable ones) needs increasing attention to protect patient privacy.

In an IoT environment, there are several challenges to securing devices and ensuring end-to-end security. Additionally, since IoT is still an emerging market, many manufacturers and designers are more concerned about launching their products to market than about designing and building security at the beginning.

One of the most frequently cited security concerns with IoT is the use of hard-coded or default passwords.

Furthermore, since a large number of IoT devices are designed to be “set and forget”—placed on a machine or the field and left at the end of their lifespans—they rarely receive security or patch updates. Adding security upfront can be costly, slow development, and prevent the device from working properly.

Another security challenge is connecting legacy assets that are not designed for IoT connectivity. It would be prohibitively expensive to replace legacy infrastructure with connected technologies. In spite of this, there are still some objects that probably have never been updated or protected against modern threats. As a result, the potential attack surface has grown.

There are also a limited number of industry-recognized standards for IoT security. Despite the existence of several IoT security frameworks, there is no single framework that has been agreed upon. 

Security and privacy must be prioritized by product manufacturers and service providers. For example, default encryption and authorization should be included.

Smart homes, connected cars, and manufacturing plants are all examples of environments that may experience IoT security breaches. For example, an attack that disables the brakes on a connected car or on a connected healthcare device can have life-threatening effects. Likewise, an attack on critical infrastructures—such as an oil well, energy grid, or water supply—could have dire consequences. Therefore, relevant industries are adopting procedures and implementing measures to ensure their safety. An analysis method that processes the data generated from all security equipment, and a measure based on previous attacks against control systems, have been developed [1].

The rise of IoT has sparked worries about the security of data transmitted between IoT devices and the edge. Indeed, Kim et al. in 2019 [2] conducted a study to address the security flaws of existing IoT devices such as sensor multi-platforms, and they proposed a model that addressed their security vulnerability.

The Elliptic/Hyperelliptic Curve (ECC/HECC) has gained increased attention in recent years because it provides shorter private key lengths with the same level of security as other PKCs such as RSA [3]. At present, cryptosystems based on asymmetric algorithms such as ECC/ECDSA are employed by many IoT devices to safeguard their data and connections [4,5,6].

In the ECC/ECDSA algorithms, protecting the private and ephemeral keys (d, k) is essential because, if an adversary obtains these keys, they could modify messages and signatures, thus making the algorithms useless. Several physical attacks aim to retrieve private and ephemeral keys (d, k) [7,8]. A side-channel attack (SCA) in cryptography is used to extract cryptographic keys and other secret information from a device such as IoT sensors, a smart card, and an integrated circuit.

The power consumption of an electronic device while processing secret data can reveal some of those details. SPA, which uses the existence of visually recognizable power consumption patterns that may expose the sequence of operations conducted by an algorithm, is one of the approaches that can be used to recover hidden information [9,10]. Hence, leakage power consumption is determined by tracking voltages of the device and then dividing by the transition count leakage and Hamming weight, with the first reflecting the number of 1-bit bits treated in time and the second reflecting the number of state variables loaded at a time [11]. Thus, if a secret value is required for this sequence of operations, the implementation is SPA-vulnerable.

Many state-of-the-art studies have examined the protection of the IoT environment against power analysis attacks [12]. Through a bit-checking mechanism, Moon et al. [13] proposed a side-channel attacks countermeasure for IoT systems. In order to eliminate branching in modulus operations, bit checking was introduced. In 2021, a statistical experimental design was proposed to optimize the power attack parameters of an IoT transducer with a minimum cost [14].

In the same year, against differential power analysis (DPA) attacks, two novel countermeasures were proposed. The two methods are based on the back-gate bias technique of fully depleted silicon on insulator (FD-SOI) technology [15]. Using the proposed countermeasures, the required number of traces to recover the secret was increased, as demonstrated by the experimental results.

Finally, SPA has been successfully implemented against a variety of cryptographic algorithms. The ECC/ECDSA key generation technique in the IoT environment is one of the algorithms that has been targeted by this type of attack.

Jérémy et al. [16] published a review on passive attacks on ECC scalar multiplication algorithms in 2016, including leakage sources and frequent errors exploited to attack the ECDSA system. This work described the link between lattice attacks and partial leakage to show how tiny leaks affect ECDSA security. In the same year, Genkin et al. [17] investigated the susceptibility of mobile devices’ ECC implementations to side-channel key extraction, finding that these implementations are vulnerable to electromagnetic and power side-channel assaults.

The authors demonstrated partial key leakage from OpenSSL running on Android and from iOS’ CommonCrypto, and complete key extraction from OpenSSL running on iOS devices. In 2018, to recover the ECDSA secret key to SM2 Digital Signature Algorithm (SM2-DSA), Zhang et al. [18] extended the new lattice-based attack introduced by Later Nguyen and Shparlinski. SM2-DSA is a Chinese version of ECDSA. They tested the security of the SM2-DSA on the Atmega128 microcontroller using a lattice attack. 

In 2019, Wunan et al. [19] published a threat analysis on the broken digital signature of data transactions and an improved SPA against ECDSA. ECDSA’s private key can be obtained by using the described attack method combined with a power trace. Moreover, Wunan et al. proposed a side-channel attacks countermeasure for blockchain devices by inserting empty operations into ECC-(point doubling/point addition) operations. In 2021, Thiebault et al. [20] presented a high-quality hardware ECDSA core and provided a complete open-source ECDSA attack artifact. They demonstrated an effective PAA against its FPGA implementation.

In ECC/ECDSA cryptosystems, the SPA leakage-based side-channel research concentrates on the modular-inversion process necessary to generate an ECC/ECDSA private key. The conversion of coordinates in an ECC implementation from projective to affine based on conventional modular inversion, for example, can lead to the disclosure of information, and an adversary could obtain the secret key. Additionally, ECDSA inverts the per-message random secret after scalar multiplication to generate a digital signature [21].

**Figure 1:**
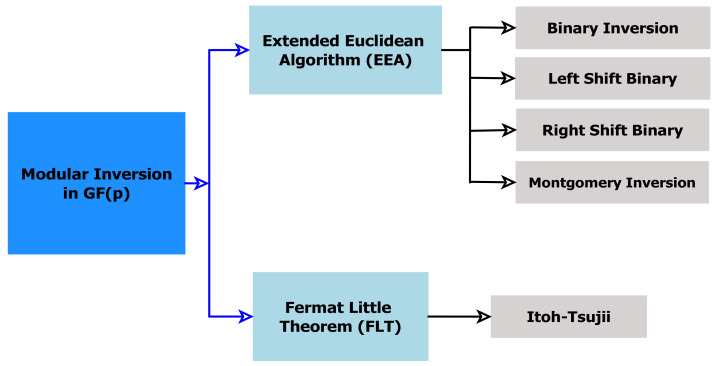
Modular inversion methods in $F_{p}$.

A challenge for cryptographic implementations is a modular inversion because it is one of the most time-consuming field operations in ECC computations. Computing modular inverses can be performed in a variety of ways. Using Fermat’s little (FLT) theorem, the Extended Euclidean algorithm (EEA), or any binary variant based on Montgomery’s Modular Inverse (MMI) algorithm, it is possible to compute the inverse function. Figure 1 shows the two popular methods, which are the FLT (its variation is the Itoh–Tsujii technique) and the EEA (its variations are the Binary Inversion Algorithm, Left Shift Binary Algorithm, Right Shift Binary Algorithm, and the Montgomery Inversion Algorithm) [22].

A comparison between the FLT, EEA, and BEEA methods is presented in Table 1. It shows that the BEEA binary version has high performance and efficiency, which meets the requirements of electronic devices (smart cards, RFID tags, mobile phones, IoT devices, etc.).

The BEEA method is commonly favored to eliminate the divisions needed in the EEA algorithm since it uses right-shift operations to replace multi-precision divisions. This improvement results in high-performance software-hardware implementations. The BEEA’s execution flow is heavily reliant on its inputs. This attribute was investigated in an attempt to find side-channel vulnerabilities that could seriously affect the privacy of a secret variable processed by BEEA.

**Figure 2:**
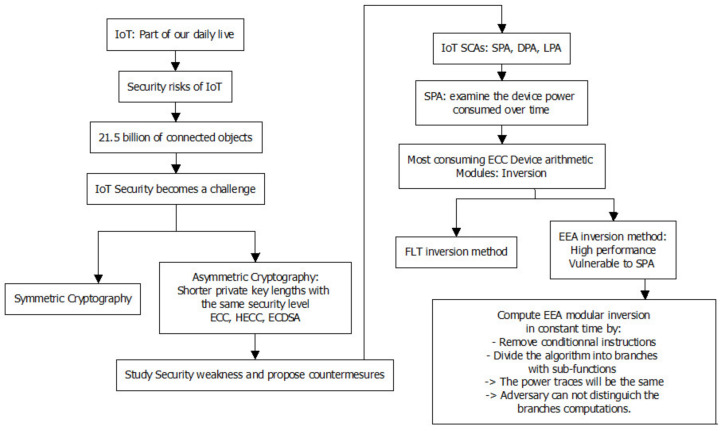
UML class diagram of IoT/modular inversion/SPA.

It is possible to leak information as a result of a typical BEEA implementation. This is because BEEA’s execution flow is heavily reliant on its inputs. Hence, the time required to calculate the result is dependent on the inputs. As a countermeasure, one of the most important elements is to ensure that the implementation has constant run-time (worst-case), meaning that the implementation’s execution time is unaffected by the input. This is usually accomplished by removing any data-dependent BEEA code branches. Hence, the main idea of this work is to compute an inverse using the same number of constant-time iterations each time. Figure 2 shows the class diagram of this work.

In addition, a high-performance modular inverter is required to speed up the calculation of a PKC system. However, key sizes grow as security levels increase, and this becomes a limiting factor in inverter design over prime field $F_{p}$ due to the carry propagation problem. As a result, adding two prime numbers (A and B) in $F_{p}$, where *p* is a prime of length > 128 bits has a direct impact on the efficiency of calculations in hardware/software (HW/SW) inverter implementations.

Over $F_{p}$, carry skip (CSK), carry select (CSL), carry-save (CSA), carry-lookahead (CLA), and parallel prefix (PPF) adders are some of the fast binary adders proposed in the literature [23]. The Kogge–Stone adder (KSA) is the fastest adder in the literature because it has a lower fan-out at each stage, which then increases its performance, and is thus widely considered as a standard adder in the industry for high-performance arithmetic circuits [24]. However, it takes more area to be implemented because it computes the carries in parallel. Therefore, KSA performs parallel additions in microprocessors, DSPs, mobile devices, and other high-speed applications.

Based on the above discussions, this paper proposes an alternative method for computing modular inversion in constant time in order to prevent SPA attacks. We modify the (non-constant-time) BEEA approach by removing the conditional instructions and dividing the algorithm into four branches with sub-functions. Since the power traces will be the same regardless of input changes, the adversary cannot distinguish the branch’s computation. Therefore, a novel BEEA-based inverter over prime fields is proposed and implemented (MBEEA).

The BEE algorithm, modular addition, and subtraction are revisited to perform MBEEA concurrently, resulting in competitive time and area complexities. Since addition is an essential operation in MBEEA, this paper introduces the generic G-KSA/S adder/subtractor to be reused for any prime number length.

The remainder of the paper is structured as follows: The (modular inversion-SPA) related works are introduced in Section 2. A brief description of BEEA is given in Section 3. Section 4 develops the constant-time BEEA-based inverter. The MBEEA architecture design is presented in Section 5. Section 6 contains the results and analysis of performance. The conclusion is given in Section 7.

## 2. Related Works

In the literature, several algorithms have been proposed for computing modular inversion. Some works were interested in analyzing SPA leakages of modular inversion implementation and their countermeasures. In 2014, to prevent combinational attacks (SPA and lattice techniques), Joppe W. Bos [25] modified the non-constant-time approach form of the Montgomery Inversion based on the EE greatest common divisor algorithm to compute both the classical and the MMI in constant time. He demonstrated that when the modulus has a special form, such as the Curve25519 (Elliptic curve with 256-bit key size offering 128-bit security), the FLT performance is comparable to the BEEA. However, the software implementation is done on ARM 32-bit, and the results showed that the constant-time almost inversion is much slower than the classic one.

The famous and easiest method to resist the leakage of information is the blinding technique using simple multiplicative masking [26]. When *Z* is the z-coordinate in projective coordinates of a given point and *u* is a random element in $F_{p}$ (unknown to the attacker), the inversion will be performed for the product *Zu* instead of directly inverting *Z* using the BEEA; finally, the result ${\left(Zu\right)}^{-1}$ is multiplied by *u* to obtain $Z^{-1}$. However, this method requires two additional modular multiplication operations. Since the modular inversion based on FLT is based on modular exponentiation, Xu et al. constructed, in 2017, a secure and efficient modular exponentiation using Montgomery friend primes described by NIST to obtain an efficient and constant-time modular inversion over $F_{p}$ [27]. Their improvement allows reducing the number of modular multiplications (a saving of 90%), which are computed using only subtractions and shifts.

In [28], the power consumption traces of two different BEEA implementations are investigated in detail in order to extract the SPA leakages that the binary EEA implementation may exhibit during the computation of the RSA key generation algorithm.

In 2018, Savaş et al. proposed two efficient constant-time Montgomery Inversion Algorithms that can be used to counter SCA [29].

The first algorithm is based on the BEEA method, and the second uses Kaliski’s method. The two algorithms have comparable performance. The number of iterations for the Montgomery Inversion Algorithm based on BEEA was 2*n* (where *n* is the bit length of modulo *p*) which is less than for the Montgomery Inversion Algorithm using Kaliski’s method (2*n* + 1). Furthermore, to speed up (upper-bounded by 2) computation in the software implementation of a multi-core processor, the author proposed a simple parallel algorithm of Montgomery Inversion.

In 2019, Bernstein et al. introduced streamlined constant-time variants of Euclid’s algorithm for polynomial and integer inputs [30]. They presented simple, fast, constant-time “division steps” that work the same for integer and polynomial inputs. After applying the main algorithm to GCD, they studied the software speed of modular inversion of polynomials as part of a key generation in the NTRU cryptosystem.

In 2021, Awaludin et al. presented a high-speed ECC processor for arbitrary Weierstrass curves over GF (p) [31]. The proposed processor works in constant time and is suitable for applications that require high speed and SCA resistance. To preserve the SCA-resistance, they used constant-time Fermat’s little theorem to perform field inversion operations.

Similarly, Sarna et al. presented a constant-time modular inversion algorithm development process capable of achieving a high level of security against timing and SPA Attacks [32].

In their work, Aldaya et al. present a novel SPA of the BEEA algorithm that reveals some exploitable power consumption-related leakages [33]. Using traces, they used the ECDSA protocol to apply SPA to reveal standardized private key sizes. An investigation was conducted into three countermeasures for eliminating SPA leakages from BEEA implementations.

## 3. BEE Algorithm

Several cryptographic algorithms use modular inverses. The BEEA is one of the most common ways to carry out the inversion operation. Given two integer numbers ‘*a*’ and ‘*p*’, BEEA computes *x* and *y* such that $ax+py=gcd\left(a,p\right)$. When $gcd\left(a,\;p\right)=1$, the calculated value for *x* refers to the multiplicative inverse of (*a* mod *p*).

The BEEA has been reported in a few different forms in the literature. The original BEEA can be shortened when it is known in advance that the modulus *p* is a prime number, as in ECDSA. Algorithm 1 illustrates the updated version of the BEEA.

According to Algorithm 1, ‘*v*-loop’ refers to the loop that divides ‘*v*’ by 2, while ‘u-loop’ refers to the loop that divides ‘*u*’ by 2.

Additionally, the term sub-step is associated with subtraction operations executed in step 5.5 to update *u* and *x* and *v* and *y*, respectively.

According to Algorithm 1, in accordance with the least significant bit (LSB) of ‘*a*’, only the u-loop during the first iteration can be executed since ‘*v*’ is equal to ‘*p*’ and ‘*p*’ is a prime number. Additionally, for the rest of the iterations, due to the subtraction at step 5.5, only one loop is executed per iteration. Hence, ‘*u*’ and ‘*v*’ are odd integers just before the execution of the sub-step stage.

A BEEA side-channel analysis was presented in [33]. It concluded that a full recovery of the algorithm’s inputs can be achieved when the adversary can collect $Z_{i}$ and SUBS[i] for all iterations, where $Z_{i}$ is the number of times that an *x*-loop (*x* = *u* or *v*) is executed at iteration ‘*i*’ and SUBS[i] is the output of the sub-step function of Algorithm 1 at iteration. When u≥v, SUBS[i] = *u*, else SUBS[i] = *v*.

**Algorithm 1:** Pseudo-code of the BEE algorithm.

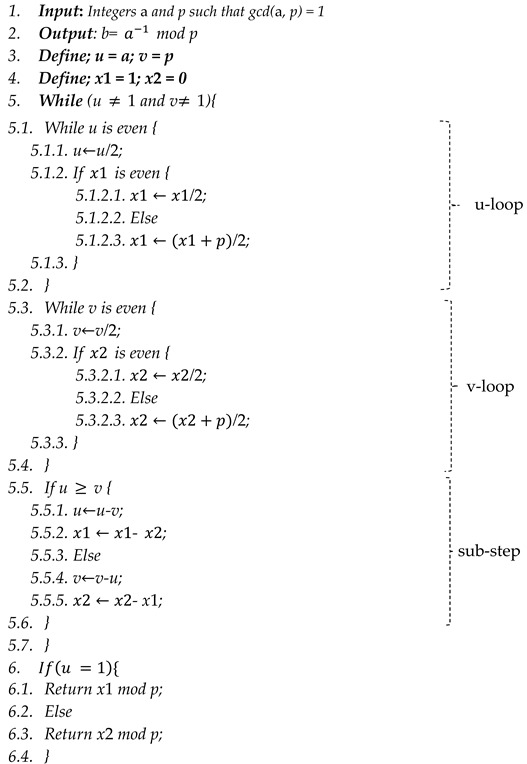



By knowing only $Z_{i}$, the authors were able to recover some SUBS[i] and to express a certain number of bits from one of the BEEA inputs as a function of the other.

It is very interesting to consider this approach in the context of power-based side-channel analysis since it appears very difficult to extract the SUBS[i] directly from power traces, while it is easier to extract the $Z_{i}$ when the sub-step can be differentiated.

We can segment BEEA iterations into x-loops followed by sub-steps. Consequently, the $Z_{i}$ is based on the duration between two consecutive sub-steps. Therefore, if an adversary can distinguish between the sub-steps of the BEEA in the power trace, then he can exploit the $Z_{i}$.

Remarkably, Algorithm 1 is based on conditional instructions (loops while and if statements). Therefore, the execution time of Algorithm 1 depends on ‘*a*’ and ‘*p*’ values. Furthermore, the input ‘*a*’ presents the scalar multiplication algorithm results in affine coordinates, and a modular inversion will convert it to projective coordinates. Hence, the inversion should be secure against SPA attacks to prevent information leakage. For this reason, our contribution is to ensure a countermeasure to prevent Algorithm 1 from a specific SPA attack.

## 4. Constant-Time Modular Inversion

Input-dependent execution flows of the BEEA have prompted the development of anti-side-channel measures. A modified version of BEEA that operates in constant time and is resistant to SPAs is proposed in this section. As a countermeasure, we propose eliminating the conditional branches in Algorithm 1 in order to reduce the data dependency.

Algorithm 1’s execution time depends on both ‘*a*’ and ‘*p*’. In such an algorithm, when different inputs are used, the number of iterations in the while loop that have to be removed at the end of the algorithm may vary significantly.

Algorithm 1 may be converted into a constant-time algorithm by meeting the following conditions:

- The same amount of time is always taken to compute an iteration. In constant time, this requires computing all four branches of Algorithm 1 and selecting the appropriate values. As a result, the computed time of each iteration is independent of the branch taken; however, it may be increased to (at most) that of the computation of the sum of all the branches.
- The algorithm always has the same number of iterations. As a result, the worst-case number of ‘*K*’ iterations should always be calculated. It can be achieved by determining when Algorithm 1 terminates (when we reach *v* = 1 or *u* = 1).

The constant-time version of Algorithm 1 is described in Algorithm 2. The first two branches are computed in every cycle (constant run-time) regardless of whether the values of *u* and *v* are even or odd. Thus, the sequence of the parity computation deduced from the power trace does not reveal any information about the inputs (the bits of a).

In Algorithm 2, the comments showing which branches from Algorithm 1 are being computed are displayed after an ‘#’. As shown in Algorithm 2, the “while loops” of Algorithm 1 are replaced by “for loops” whose higher bound is ‘*K*’. ‘*K*’ presents the cycle number of the biggest number inverse in the finite field $F_{p}$, such that (*a* < *p*). Therefore, *K* presents the worst case of iterations.

It is not difficult to determine the number of iterations (‘*K*’) in the worst-case scenario of Algorithm 1. In every iteration, either ‘*u*’ or ‘*v*’ are reduced by at least a factor of two, so the maximum number of iterations is $2log_{2}\left(p\right)$, where *p* is a prime number of n-bit length. It follows that the minimum number of iterations is $log_{2}\left(p\right)$. This reveals the bounds on the exponent ‘*k*’ when the algorithm terminates. The functions presented in Algorithm 2 are:

- The shift-by-one function denoted by ${\mathrm{lshift}}_{1}\left(z,x\right)$: this function shifts *x* by one position to the left and stores the result in *z*.
- The subtraction and addition functions are denoted respectively by sub (*z*, *x*, *y*) and add (*z*, *x*, *y*)) computing *z* ← *x* − *y* and *z* ← *x* + *y*. Those two functions are computed by the adder/subtractor G-KSA/S which is discussed in the next section.

For the function add ($x_{1}$, $x_{1}$,(*p*’ $\wedge {\;x}_{1}$ (0)), we create a bitmask $x_{1}$(0) since the LSB of $x_{1}$ determines the parity of $x_{1}$ (1 indicates odd, 0 indicates even) in order to calculate the iteration 5.1.2 in Algorithm 1.

(1) add (x1, x1,(p′ ∧ x1 (0)) =  if  x1 is even then  x1 0 =0→ x1=x12if  x1   is odd then  x1 0 =1→ x1=x12+p′

- The function $mem_{u}\;and\;mem_{v}$ are used respectively to save the values of *u* and $x_{1}$ if *u* is even ($mem_{u}$ = 0, RAM active 0), and the values of *v* and $x_{2}$ if *v* is even ($mem_{v}$ = 0, RAM active 0). As the LUT area is 2*n*, we need four look-up tables to memorize *u*, $x_{1}$, *v*, and $x_{2}$.
- The comparison function is denoted by comp (*u*, *v*). This function is used to compare ‘*u*’ and ‘*v*’ values.
  (2) $$\left\{\begin{array}{c} if\;u\geq v\;\mathrm{and}\;\;u\neq 1\;\;\mathrm{then}\;\;sel_{1}=1,\;else\;sel_{1}=0\;\\ if\;v\geq u\;\mathrm{and}\;u\neq 1\;\mathrm{then}\;sel_{2}=1,\;else\;sel_{2}=0 \end{array}\right.$$
- The function denoted $mem_{u}$($sel_{1}$)
  (3) $$\left\{\;\begin{array}{l} \;if\;sel_{1}=1\;\mathrm{then}\;u\;\mathrm{and}\;x_{1}\;\mathrm{alookuped}\;\mathrm{in}\;\mathrm{the}\;\mathrm{look}\;\mathrm{up}\;\mathrm{tables}\\ else\;no\;values\;are\;stored \end{array}\right.$$
- The occurrence function is denoted by occur (RAM, *u*, 1, *i*), which means the first occurrence of 1 in RAM of *u* indicates the corresponding *i*, then:
  (4) $$\left\{\begin{array}{l} \;If\;i<j\;\;\;\;then\;\;\;b=x_{1}\\ \;If\;j>i\;\;\;\;then\;b=x_{2}\; \end{array}\right.$$

**Algorithm 2:** Pseudo-code of the proposed modified BEE algorithm*1*. **Input:***p* and *a* ∈ $F_{p}$
*2*. ***Output***$:\;b=a^{-1}$
*mod p*
*3*. ***Define; *u* = *a*; *v* = *p****
*4*. ***Define; *x*1 = 1; *x*2 = 0***
*5*. ***Define;** p’ = Rshift(*p*,1)*
***Inversion** steps*
*6*. ***for*** (i=1; i≤k; *i++) {*
  *6.1.* $lshift_{1}\left(u,u\right)$
  *6.2.* $lshift_{1}\left(x_{1},x_{1}\right)$
  *6.3.* *add(*$x_{1}$, $x_{1}$,(*p’*
$\wedge \;x_{1}$
*(0))*
  *6.4.* $mem_{u}$
*(u(0))*
# *u*$\leftarrow \;SHIFT\left(u,1\right)$ #$x_{1}\leftarrow SHIFT(x_{1},1)$ #$x_{1}\leftarrow \mathrm{SHIFT}(\mathrm{ADD}(x_{1},p),1)$

  *6.5.* $lshift_{1}\left(v,v\right)$
  *6.6.* $lshift_{1}\left(x_{2},x_{2}\right)$

  *6.7.* *add($x_{2}$, $x_{2}$,(p’ $\wedge \;x_{2}$ (0))*
  *6.8.* $mem_{v}$
*(v(0))*
# *v*$\leftarrow SHIFT\left(v,1\right)$ #$x_{2}\leftarrow SHIFT(x_{2},1)$ #$x_{2}\leftarrow \mathrm{SHIFT}(ADD(x_{2},p),1)$

  *6.9.* *comp(u,v)*
  *6.10.* $sub\left(u,u,v\right)$ $\wedge sel_{1}$
  *6.11.* $sub(x_{1},x_{1},$ $x_{2})\wedge sel_{1}$# If *u*
≥
*v* # *u*$\leftarrow \;SUB\;\left(u,v\right)$ #$x_{1}\leftarrow SUB\;(x_{1},$ $x_{2})$

  *6.12.* $mem_{u}$ ($sel_{1}$)
  *6.13.* $sub\left(v,v,u\right)$ $\wedge sel_{2}$
  *6.14.* $sub(x_{2},x_{2},$ $x_{1})\wedge sel_{2}$
  *6.15.* $mem_{v}$ ($sel_{2})$# *v*$\leftarrow SUB\;\left(v,u\right)$ #$x_{2}\leftarrow SUB\;(x_{2},$ $x_{1})$

  *6.16.* *occur(RAM, u, 1,i)*

  *6.17.* *occur(RAM, v, 1,j)*

  *6.18.* *return (i,j,x)* *7.* *}*# Return $x_{1}$ mod *p* if *u* equal 1 # Return $x_{2}$ mod *p* if *v* equal 1

Overall, Algorithm 2 in $F_{p}$ has two specified inputs: an element ‘*a*’ and a prime number ‘*p*’. The output of the algorithm is the multiplicative inverse of ‘*a*’, i.e., ba=1 mod p. Algorithm 2 employs three steps in total to produce the multiplicative inverse result *p*:

- The initial step: ‘*a*’ and ‘*p*’ are assigned to ‘*u*’ and ‘*v*’, respectively. Moreover, in this step, ‘1′ and ‘0′ are assigned to *x*1 and *x*2, respectively.
- Updating step: *u*, *v*, *x*1, and *x*2 values are updated by three different operations: add, sub, left shift (lshift), $mem_{u}$, and $mem_{v}$. The updating process is executed throughout the entire loop ((1 < *i* < *k*) execution time) and managed by two signals: sel1 and sel2. The new (*u*, *v*, *x*1, and *x*2) values are assigned according to sel1 and sel2, and these control signals are updated in step 12.9 during each iteration of the “for loop”.
- Return ‘*b*’ after *k* iterations.

## 5. MBEEA Implementation

As shown in Algorithm 2, inversion is realized mainly by subtracting and shifting operations. Additionally, shift operations are easily implemented in hardware at no cost. We can therefore consider in our design a case that allows performing as many shifts as possible in one clock cycle to reduce the number of clock cycles. 

As seen in Algorithm 2, ‘Reg_u’, ‘Reg_v’, ‘Reg x1′, and ‘Regx2′ *n*-bit registers are essential for implementing the hardware architecture of MBEEA over a prime field. By applying this algorithm, the calculation of division, such as ‘*u*/2′, ‘*v*/2′, ‘*x*1/2′, and ‘*y*1/2′, is based on comparisons of parity and magnitude. There are two multiplexers for selecting ‘*u*’, ‘*v*’, ‘*x*1′, and ‘*x*2′. However, exact comparisons can only be made through full *n*-bit subtractions, and this has the effect of delaying decisions about the next calculation. To perform the additions or subtractions, we used *n*-bit KSA. In the implemented design, all possible ‘*u*’, ‘*v*’, ‘*x*1′, and ‘*x*2′ updated values are computed at once with multiplexers, and the new values for ‘*u*’ and ‘*v*’ are selected.

**Figure 3:**
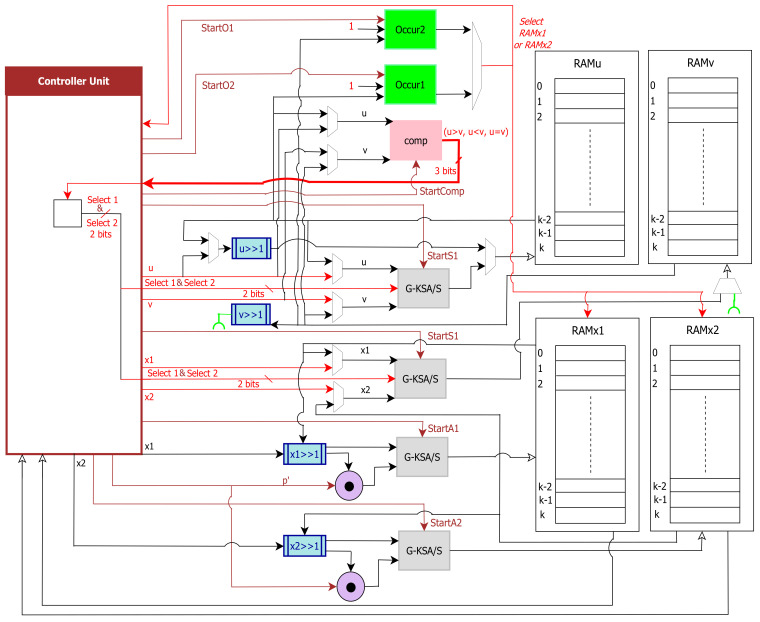
Proposed MBEEA architecture.

Figure 3 depicts the proposed MBEEA inverter design, which is based on Algorithm 2 and includes the following units:

- *Controller unit:* This generates control signals for all the MBEEA architecture units and the data flow in the inverter design, and the movement of data between the adder-subtractor units, the memory units, the comparator unit, and the occurrence units.
- *Adder/Subtractor (G-KSA/S):* This performs the addition or the subtraction of two values according to the controller decision.
- *Memory unit (MU):* The main purpose of this unit is to store different parameters such as *u*, *v*, *x*1, and *x*2, and their intermediate results. It constitutes of four blocks of RAM (RAMu, RAMv, RAMx1, and RAMx2) and multiplexers that are used to read operands (*u*, *v*, *x*1, and *x*2) from the MU using the corresponding control signals.
- *Comp unit:* This is a comparator between *u* and *v*. It has a 3-bit output to indicate whether *u* > *v* or *u* < *v* or *u* = *v*.
- *Occur unit:* This searches for *u* = 1 occurrence. Return *x* 1 mod *p* if *u* equal 1. Otherwise, if *v* is 1, return *x*2 mod *p*.

### 5.1. Kogge–Stone Adder/Subtractor Unit: Design and Implementation

Adders are widely recognized as the fundamental building blocks for more complex arithmetic operators such as multipliers and inverters. Usually, subtraction and addition are implemented using a single circuit. The addition of *x*, $\bar{y}$, and 1 can be used to compute the subtraction of *x* − *y*, where *y* is the bitwise complement of *y*. Therefore, hardware addition can be used to support the subtraction function. To perform the addition and subtraction in Algorithm 2, four adders/subtractors are needed. The data path of the adder directly affects the delay of the arithmetic inversion data path. KSA is the fastest adder and shows good hardware performance. Thus, KSA was selected for use in the MBEEA architecture for performing addition and subtraction. A parallel prefix adder such as KSA is suitable for additions with longer word lengths. The tree network of KSA allows reducing the latency to $O\left(log_{2}n\right)$ where ‘*n*’ represents the number of bits.

The KSA algorithm is composed of three stages: preprocessing, parallel prefix network, and post-processing. In the preprocessing stage, two signals are calculated simultaneously for each input: Propagate (*P*) and Generate (*G*). The intermediate carries are then generated using these signals in the parallel prefix network. Finally, in the post-processing, we calculate the sum by XORing the intermediate carries and the propagate signals.

In the MBEEA design, a generic adder/subtractor based on the G-KSA/S algorithm was developed. The bit-level version of the G-KSA/S algorithm is shown in Algorithm 3, which employs n-bit registers to compute three intermediate results *P*, *G*, and C. Overall, Algorithm 3 has two specified inputs: A and *B* in $F_{p}$ and a pre-computed vector *S*. The pre-computed vector *S* is represented by a set of elements ‘*s*_*i*_’= 2^*i*^ where $i\in \left[0,\;m-1\right]$, and *m* represents the stage number which depends on the length of the prime number *p* (*n*), such that $m=log_{2}\left(n\right)$. Table 2 presents the ‘*m*’ and ‘*S*’ values for a prime number of lengths *n* = 4, 8, 16, 32, 64, 128, and 256. Hence for *m* = 3, *S* = {*s*_0_, *s*_1_, *s*_2_} = {2^0^, 2^1^, 2^2^}.

*A* and *B* in $F_{p}$, two *n*-bit inputs, are processed in the first stage bit-by-bit, to compute the generation signals $G_{0}$ and propagation signals $P_{0}$ which are presented in steps 2.1 and 2.2 of Algorithm 3. To perform the subtraction, the *carry_in* and the input *B* are just XORed, such as presented in step 1 in Algorithm 3.

**Algorithm 3:** G-KSA/S algorithm
Input: (*A*,*B*) ∈
$F_{p}$
Define: *n; n = bit length of p* Pre-computed:*S* = [1,2,4,8,16,32,64,128], *j*
←1, *k*← 1
Output: ($Sum=\left(A+B\right)mod\;p;\;c_{out})$
Preprocessing
**Step 1:** For (*i* = 0; *i*≤ to *n* − 1; *i*++) {
C(*i*) := $c_{in}⊕$*B*(*i*);
};
**Step 2:** For (*i* = 0; *i*≤
*n* − 1; *i*++) {
2.1:$P_{0}\left(i\right)$ := *A*(*i*)⊕*C*(*i*);
2.2:$G_{0}\left(i\right)$ := *A*(*i*)⊗*C*(*i*);
};
Parallel Prefix Network
**Step 3**: For (*i* = 0; *i*≤ *j*-1; *i*++) {
3.1$:G_{k}\left(i\right)$ := $G_{k-1}\left(i\right)$,
3.2$:P_{k}\left(i\right)$ := $P_{k-1}\left(i\right)$
**};**
**Step 4:** For (*i* = 0; *i*≤n−*j*-1){
4.1:$G_{k}\left(i+j\right)$ := ($P_{k-1}\left(i+j\right)⊗\;G_{k-1}\left(i\right)))\vee G_{k-1}\left(i+j\right)$;
4.2: $P_{k}\left(i+j\right)$ := ($P_{k-1}\left(i+j\right)⊗;P_{k-1}\left(i\right))$;
**};**
*k* + +;*j* := *S*[*k*-*1*], T := *m* − 1;
**IF(*T* > 0) go to step 3 ELSE go to step 5**
**Step 5:** For (*i* = 0; *i*
≤ *n*-1; *i*++) {
5.1: *C*(*i*) := $G_{NP-1}$(*i*)∨($c_{in}⊗P_{NP-1}$(*i*));
**};**
Post-processing
**Step 6:** 6.1:sum(0) = $P_{0}$(0) $⊕\;c_{in}$;
6.2: For (*i* = 1; *i* ≤ *n*-1; *i*++){
6.2.1: sum(*i*) = $P_{0}$(*i*)⊕*C*(*i* − 1);
**};**
**Step 7:**
$c_{out}$ = *C*(*N* − 1);
Return Sum, $c_{out}$


**Figure 4:**
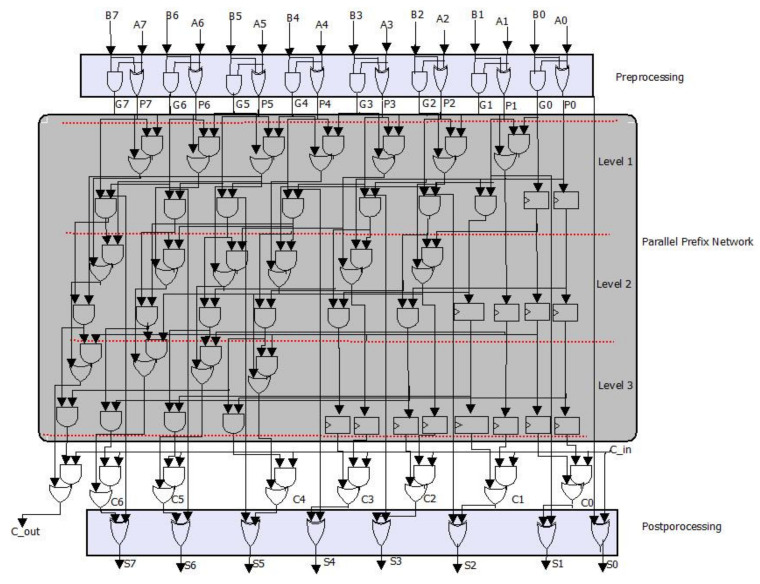
Eight-bit G-KSA/S data-path.

Figure 4 presents the KSA process for 8 bits. As shown in Figure 3, the parallel prefix network for G-KSA/S is composed of three levels. The carry propagation network is in charge of transmitting the carry signal from the preceding bit lines. $P_{k}$ and $G_{k}$ are generated via ‘carry propagation’ steps, steps 4.1.1 and 4.1.2, as shown in Algorithm 3. Finally, the carry signal obtained in step 5 is used to calculate the sum in the last stage (step 6). The output step performs the XOR operation between the previous bits of the ‘carry’ signal (c_i−1_) and the current bits of the propagation signal (Pi), as shown in steps 6 and 7.

### 5.2. The Modular Reduction

The modular reduction in large values is a fundamental operation in the majority of common PKs that involve intensive computations in prime fields. At the end of Algorithm 2, the result will be reduced modulo the prime number ‘*p*’. The prime number should be chosen carefully to ensure efficient reduction and high performance. The modular reduction can be calculated in three ways:

- General module forms (i.e., Barrett and Montgomery algorithms), which are slower and present an expansive part of the arithmetic operation.
- Based on the LUT method (which is based on pre-computed values); however, this requires a large amount of memory.
- Modulo form of prime numbers, such as pseudo-Mersenne numbers. Their special form makes them appropriate for modular reduction. The pseudo-Mersenne prime number *p* is presented with the special form: *p* = $2^{\alpha }$ – *c*, where α = *n* (*n* represents the security level) and ‘*c*’ is a positive integer that is relatively small compared to the modulus. An integer $0\leq z<{\left(2^{\alpha }-c\right)}^{2}$ can be represented in radix-$2^{\alpha }$ by spilling z up into a lower part $Z_{L}$ and a higher part $Z_{H}:{\;Z}_{\;H}2^{\alpha }$ + $Z_{L}$. Then, using the fact that $2^{\alpha }$ ≡ c mod p, we have $Z_{H}2^{\alpha }$ + $Z_{L}$ ≡ $Z_{H}$c + $Z_{L}$ mod *p* where 0 ≤ $Z_{H}$c + $Z_{L}$ < (c + 1)$2^{\alpha }$. Hence, a multiplication of $Z_{H}$ by the constant c is needed and the final result is obtained after the addition of $Z_{L}$. For the three values of *n* (128, 192, and 256), the resulting primes satisfy *p* ≡ 3 (mod 4).

### 5.3. Controller Unit

The main component of the proposed inverter design is the control unit (CU), which is in charge of all communications between all MBEEA units.

**Figure 5:**
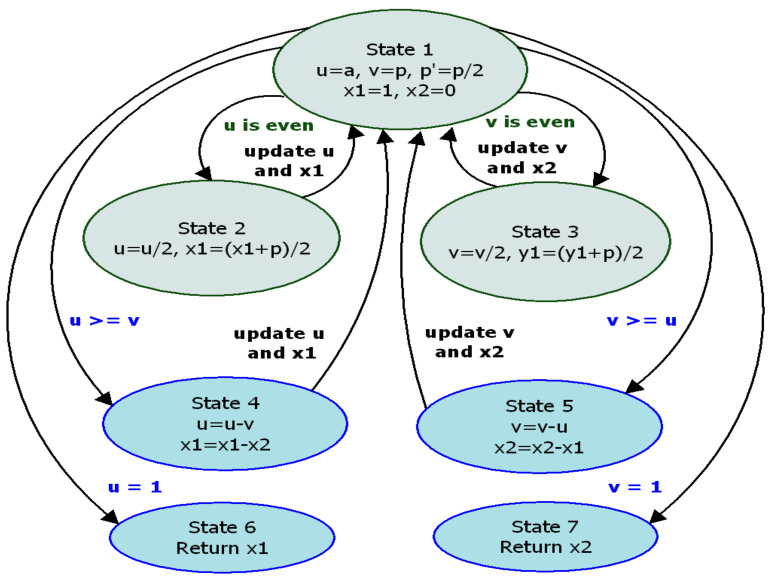
MBEEA state machine.

At the beginning of the algorithm, the start signal is equal to ‘1’, and initial values ‘a’, ‘*p*’, ‘1’, ‘0′, and ‘*p*/2’ are assigned to ‘*u*’, ‘*v*’, ‘*x*1’, ‘*x*2’, and ‘*p*’ registers, respectively, by input multiplexers. The computed intermediate results are stored in the memory units. The control unit activates the addition/subtraction chains after receiving the input data (*p* and *a*) and the signals “CLK”, “Reset” = ‘1’, and “Start” = ‘1’. Hence KSA-start takes ‘1’ and Cf becomes ‘0’. If KSA completes the addition function (“KSA-done” = ‘1’), the control unit sends signals of writing to the memory unit to update the ‘*u*’, ‘*v*’, ‘*x*1’, and ‘*x*2’ values. The update process is controlled by five functions belonging to three blocks: addition and subtraction functions (KSA block), left shifting function (shift registers), writing, and reading functions (block memory). These new values are determined by two distinct control signals: sel1 and sel2. These control signals are updated after the KSA block performs the ‘*u*-*v*’ function as shown in step 12.9 in Algorithm 2. Hence, the CU generates the signals for the G-KSA/S components, and the read and write addresses for the memory units. The MBEEA state machine describing the design data flow is presented in Figure 5. 

To implement the MBEEA, FSM is made up of seven states: St#1 is a state of inactivity, whereas during St#2 to St#7, necessary signals for updating ‘*u*’, ‘*v*’, ‘*x*1’, and ‘*x*2’ are generated. Of these seven states, two states (S#2, S#3) are executed in parallel. Hence two additions, four shifting operations, and four reading/writing operations are required. Similarly, in states 4 and 5, two subtractions and two reading/writing operations are needed. Finally, based on the output of the comparator unit, ‘*u*’ and ‘*x*1’ or ‘*v*’ and ‘*x*2’ are updated.

## 6. Results and Performance Analysis

The proposed MBEEA inverter and G-KSA/S designs were then developed for a different prime field of lengths 8, 16, 32, 64, 128, and 256 bits. Modelsim was used to validate the proposed designs, which were coded in VHDL. The G-KSA/S and MBEEA designs were then implemented on various devices (Spartan 3E, Virtex-E, Virtex-II, Virtex 5, Virtex 7) using Xilinx 14.7. Table 3 and Table 4 show the obtained results after place and route, which include the maximum frequency (Fmax, MHz), area usage (slices/CLB), latency (μs), and ADP (ADP = #slices × Latency).

### 6.1. G-KSA/S Implementation Results

**Figure 6:**
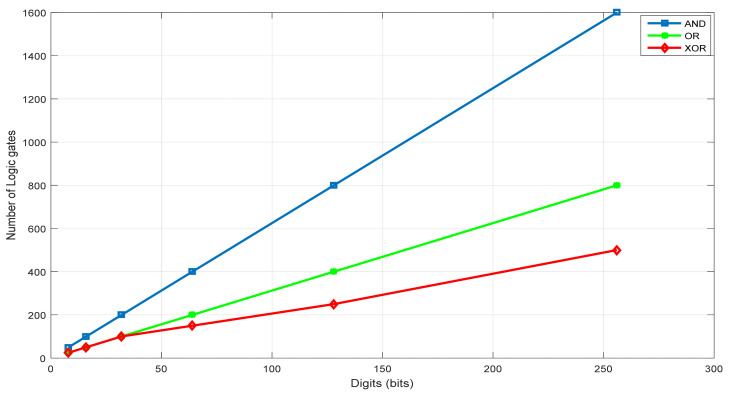
Logic gates versus $F_{p}$.

The G-KSA/S algorithm was successfully implemented for $F_{p}$ (*p* is a prime number of length 8, 16, 32 64, 128, 256 bits). Figure 6 presents the required logic gates (AND, OR, and XOR) for the GKSA design (for G-KSA/S, we have to add one XOR gate for subtraction). We can see that the number of required logic gates grows linearly.

For a fair comparison with related works, the G-KSA/S algorithm was implemented on Spartan3E and Virtex-5, as shown in Table 3. As shown in Table 3, the proposed design clearly outperforms the existing different prime field-length KSA implementations. We can see from Table 3 that our proposed design is much smaller (almost 66% for $F_{p}$ (*p* is a prime of length 8, 16, and 32), and almost 41% for $F_{p}$ (*p* is a prime of length 64, 128, and 256)) and considerably faster than those of [34,35].

Furthermore, the G-KSA/S proposed design always achieves the best ADP value in all the prime fields compared to the existing designs. For example, over $F_{p}$ with a length of *p* = 256 bits, compared with [35] on the Virtex-5 device, the ADP of the proposed design has a 28.62% smaller ADP.

**Table 3:** Comparison of the proposed and existing G-KSA/S designs for different prime field lengths.

Designs	Platform	*n* = Bit Length of *p*	Area (Slices)	Delay (ns)	ADP (10^−9^)	Gain %
[34]	Spartan-3E	8	83	5.776	479.408	
G-KSA/S	Spartan-3E		47	3.6	169.2	74.71%
[34]	Spartan-3E	16	166	10.85	1801.1	
G-KSA/S	Spartan-3E		98	7.3	715.4	61.28%
[34]	Spartan-3E	32	332	20.56	6825.92	
G-KSA/S	Spartan-3E		174	12.3	2140.2	68.65%
[35]	Virtex-5	64	449	30.5	13,694.5	
G-KSA/S	Virtex-5		289	27.9	8063.1	41.13%
[35]	Virtex-5	128	1111	57.3	63,660.3	
G-KSA/S	Virtex-5		641	64.4	41,280.4	35.16%
[35]	Virtex-5	256	1345	106.7	143,511.5	
G-KSA/S	Virtex-5		737	139	102,443	28.62%

### 6.2. MBEEA Implementation Results

Table 4 summarizes the MBEEA hardware implementation results on a contemporary Xilinx Virtex-7 (x7vx330t-2.g1157) FPGA. The proposed MBEEA generic architecture designs were implemented over different prime field lengths (*n* (bit length of *p*) = 8, 16, 32, 64, 128, and 256 bits). In practice, 128- and 256-bit lengths for ECC/HECC and pairings systems, over prime fields, are very useful for modern security applications. The post place and route-static timing report was used to calculate the minimum clock period.

**Table 4:** FPGA implementation performance for the proposed MBEEA design in Virtex-7.

Design	n = Bit Length of *p*	Freq. (MHz)	Area (Slices)	Latency (μs)
	8	530	545	0.179
	16	480	770	0.27
MBEEA	32	420	1060	0.346
	64	380	1237	0.428
	128	310	1532	0.851
	256	250	2035	1.24

In Table 5, we compare the corresponding FPGA implementation results with those of reports available in the literature to additionally assess the actual performance of the proposed inverter design over $F_{256}$. $F_{p}$ for a prime of length 256 bits is considered to be the large prime field size. Table 5 lists all related performance metrics: area, frequency (MHZ), latency (μs), and ADP. As an overall performance metric, we used ADP for all related designs to ensure a fair comparison.

Table 5 clearly shows that the proposed design outperforms the existing large prime field-size modular inversion implementations.

In terms of latency, on the Virtex-7 device, the proposed design is 51.2% and 54.29% faster than those of [36,41], respectively. At the same time, on the Virtex-6 device, the proposed design is 61% and 63.35% faster than the competing designs of [38,39], respectively. On the Virtex-II, the proposed design remarkably outperforms all the existing designs of [10,42,43,44,45,46] in terms of latency and area. For instance, the works presented in [43,46] utilize 72.54% and 53.81%, respectively, more FPGA slices than this work. 

At the same time, Table 5 shows that the proposed design ensures significantly lower ADP than the existing designs. Compared with [36,37,41] on the Virtex-7 device, the ADP of the proposed design has 23.81%, 23%, and 41.18% smaller ADP, respectively.

## 7. Conclusions

Securing the IoT is one of the major, if not the major, challenges for IT systems today. If the IoT is not sufficiently protected, critical events can occur, such as water or power outages, or worse, manipulated processes resulting in bacteria in water and faulty products such as cars, etc., that pose security risks. This urgent need for security, combined with the lack of security of IoT devices that are simultaneously connected to the Internet, which has a high risk of threat, illustrates the importance of the topic. The data and connections of many IoT devices are secured by cryptographic algorithms such as ECC and ECDSA. ECC/ECDSA is one of the algorithms targeted by the SPA attack. The modular BEEA inversion process used for generating an ECC/ECDSA private key is the focus of the leakage-based side-channel study. During the execution of the cryptographic algorithm, SPA can easily distinguish the conditional branches outcomes since a device consumes power differently and the execution time is not constant.

A new version of the BEE inversion algorithm was proposed that works in constant time by avoiding the data dependency of the classical BEEA. To ensure the traces are the same, thus preventing an attacker from distinguishing the computation across branches, the proposed method removes conditional instructions and divides the algorithm into separate branches with sub-functions. The classical BEEA is secured against SPA attacks due to the new countermeasure.

The new algorithm was designed so that the calculation of the modular inversion is easy and efficient for hardware implementation. The algorithm complexity is suitable even for a device having limited resources such as an IoT device. To achieve high performance on an FPGA, a variety of optimization techniques, including algorithmic reformulation and architectural optimization, are used. On a Xilinx Virtex-7 FPGA, our design can achieve a maximum clock rate of 276 MHz and it takes only 1.12 μs to perform the 256-bit prime modular inversion.

The proposed design for $F_{256}$ provides an ADP figure of 2.28 on a Virtex-7, which is less than the relevant state-of-the-art solutions. Furthermore, our architecture outperforms others in terms of FPGA area (slices) and delay. Therefore, the proposed design is suitable for constrained implementations of cryptographic primitives, such as IoT, wireless sensor nodes, and RFID devices.

## Figures and Tables

**Table 1:** Comparison between FLT, EEA, and BEEA methods.

FLT	EEA	BEEA
More complicated than EEA $\mathrm{Higher}\;\mathrm{complexity}\;O\;(log^{3}$ n) Slower (consumes a lot of time compared to EEA) Uses a large number of repetitive multiplications Secure against SPA and timing attack	More efficient than FLT $\mathrm{Less}\;\mathrm{complexity}\;O\;(log^{2}$ n) Very fast and commonly used for large operands	Suitable for hardware implementation because it replaced expensive divisions with shift-right operations Faster than EEA Less complicated (uses only ordinary additions and subtractions) No need for multiplications or divisions

**Table 2:** Values of *m* and *S* for *n* = 4, 8, 16, 32, 64, 128, and 256 bits.

*n*	*m*	*S*
4	2	1,2
8	3	1,2,4
16	4	1,2,4,8
32	5	1,2,4,8,16
64	6	1,2,4,3,16,32
128	7	1,2,4,3,16,32,64
256	8	1,2,4,3,16,32,64,128

**Table 5:** Performance analysis of the proposed and the existing modular inversion designs over F_256_.

Ref.	FPGA Device	Freq (MHz)	Time (μs)	Area (Slices)	ADP (×10^−9^)
[36]	Virtex-7	146.23	2.329	1480	3.44
[37]	Kintex 7	142.38	2.33	1480	3.45
[38]	Virtex-6	151	3.39	1190	4.04
[39]	Virtex-6	146	3.52	1340	4.72
[40]	Virtex-5	129	7.937	592	4.7
[41]	Virtex-7	138.3	2.45	1577	3.87
[42]	Virtex-II	55.70	6.2	5863	36.35
[43]	Virtex-II	37	4.98	9213	45.88
[44]	Virtex-II	68.17	11.60	2085	24.19
[10]	Virtex-II	34	14.6	9146	133.53
[45]	Virtex-II	40.68	15.22	14,844	225.26
[46]	Virtex-II	50	6.4	5477	35
MBEEA	Virtex-E	106	2.92	2830	8.26
MBEEA	Virtex-II	175	1.77	2530	4.47
MBEEA	Virtex-5	208	1.49	2318	3.45
MBEEA	Virtex-6	240	1.29	2140	2.76
MBEEA	Virtex-7	276	1.12	2035	2.28
